# Phytochemical Profile and Herbicidal (Phytotoxic), Antioxidants Potential of Essential Oils from *Calycolpus goetheanus* (Myrtaceae) Specimens, and in Silico Study

**DOI:** 10.3390/molecules27154678

**Published:** 2022-07-22

**Authors:** Celeste de Jesus Pereira Franco, Oberdan Oliveira Ferreira, Jorddy Neves Cruz, Everton Luiz Pompeu Varela, Ângelo Antônio Barbosa de Moraes, Lidiane Diniz do Nascimento, Márcia Moraes Cascaes, Antônio Pedro da Silva Souza Filho, Rafael Rodrigues Lima, Sandro Percário, Mozaniel Santana de Oliveira, Eloisa Helena de Aguiar Andrade

**Affiliations:** 1 Faculdade de Química, Universidade Federal do Pará, Rua Augusto Corrêa S/N, Guamá, Belém 66075-900, Pará, Brazil; celeste.frango12@gmail.com (C.d.J.P.F.); angeloquimica17@gmail.com (Â.A.B.d.M.); 2 Programa de Pós-Graduação em Biodiversidade e Biotecnologia-Rede Bionorte, Instituto de Ciências Biológicas, Universidade Federal do Pará, Rua Augusto Corrêa S/N, Guamá, Belém 66075-900, Pará, Brazil; oberdan@museu-goeldi.br (O.O.F.); evertonlpvalerla@gmail.com (E.L.P.V.); eloisa@museu-goeldi.br (E.H.d.A.A.); 3 Laboratory of Functional and Structural Biology, Institute of Biological Sciences, Universidade Federal do Pará, Rua Augusto Corrêa S/N, Guamá, Belém 66075-900, Pará, Brazil; jorddy.cruz@icb.ufpa.br (J.N.C.); rafalima@ufpa.br (R.R.L.); 4 Laboratório de Pesquisas em Estresse Oxidativo, Instituto de Ciências Biológicas, Universidade Federal do Pará, Rua Augusto Corrêa S/N, Guamá, Belém 66075-900, Pará, Brazil; percario@ufpa.br; 5 Laboratório Adolpho Ducke—Coordenação de Botânica, Museu Paraense Emílio Goeldi, Av. Perimetral, 1901, Terra Firme, Belém 66077-830, Pará, Brazil; lidianenascimento@museu-goeldi.br; 6 Programa de Pós-Graduação em Química, Universidade Federal do Pará, Rua Augusto Corrêa S/N, Guamá, Belém 66075-900, Pará, Brazil; cascaesmm@gmail.com; 7 Embrapa Amazônia Oriental, Tv. Dr. Enéas Pinheiro, s/n-Marco, Belém 66095-903, Pará, Brazil; antoniopedro.filho@embrapa.br

**Keywords:** natural products, volatile compounds, terpenes, allelopathy, antioxidant capacity

## Abstract

The essential oil (EO) of Calycolpus goetheanus (Myrtaceae) specimens (A, B, and C) were obtained through hydrodistillation. The analysis of the chemical composition of the EOs was by gas chromatography coupled with mass spectrometry CG-MS, and gas chromatography coupled with a flame ionization detector CG-FID. The phytotoxic activity of those EOs was evaluated against two weed species from common pasture areas in the Amazon region: *Mimosa pudica* L. and *Senna obtusifolia* (L.) The antioxidant capacity of the EOs was determined by (DPPH^•^) and (ABTS^•+^). Using molecular docking, we evaluated the interaction mode of the major EO compounds with the molecular binding protein 4-hydroxyphenylpyruvate dioxygenase (HPPD). The EO of specimen A was characterized by β-eudesmol (22.83%), (*E*)-caryophyllene (14.61%), and γ-eudesmol (13.87%), while compounds 1,8-cineole (8.64%), (*E*)-caryophyllene (5.86%), δ-cadinene (5.78%), and palustrol (4.97%) characterize the chemical profile of specimen B’s EOs, and specimen C had α-cadinol (9.03%), δ-cadinene (8.01%), and (*E*)-caryophyllene (6.74%) as the majority. The phytotoxic potential of the EOs was observed in the receptor species *M. pudica* with percentages of inhibition of 30%, and 33.33% for specimens B and C, respectively. The EOs’ antioxidant in DPPH^•^ was 0.79 ± 0.08 and 0.83 ± 0.02 mM for specimens A and B, respectively. In the TEAC, was 0.07 ± 0.02 mM for specimen A and 0.12 ± 0.06 mM for specimen B. In the results of the in silico study, we observed that the van der Waals and hydrophobic interactions of the alkyl and pi-alkyl types were the main interactions responsible for the formation of the receptor–ligand complex.

## 1. Introduction

In the last decades, EOs have been applied in several industry sectors, among which are the cosmetics, pharmaceutical, and food industries, being used primarily as food flavoring, medication, and in the preparation of fragrances [1,2,3,4,5]. Aromatic and medicinal plants produce EOs that are recognized for their aroma and flavor characteristics, and their antioxidant and biological properties such as: antimicrobial, anticancer, and cytotoxic [2,6].

The biological properties of EOs are strongly influenced by their chemical composition [7], containing complex mixtures of volatile and low-molecular-weight organic compounds. Within the composition of EOs, there are several chemical structures that encompass two groups with distinct biosynthetic origins: terpenes (monoterpenes and sesquiterpenes) and another group of aliphatic and aromatic compounds (for example, aldehydes, phenols, among others) [8]. Within this context, we can highlight that the Amazon region is a source of species rich in EOs, among which are species of Myrtaceae that are widely distributed in the tropical and subtropical regions of the planet [9,10].

The Myrtaceae family is comprised of approximately 132 genera and over 6000 tree and shrub species [10,11,12], and in Brazil, we can find 29 genera and 1192 species [13]. Recent studies show that EOs from the Myrtaceae family have a great potential to solve problems in several industries, such as health, food, and even agricultural production [14], as they have important properties, including antioxidant, insecticide, parasiticide, antimicrobial [14,15]. 

Studies highlight Myrtaceae as a source of compounds of biological interest against pests or pathogens due to its essential oil content [16,17]. With regard to the herbicidal properties of EOs, the search for alternative sources of natural origin to replace synthetic herbicides is increasing nowadays, as the excessive use of synthetic herbicides causes serious damage to human health and the environment, due to their high toxicity and low biodegradability [18]. Moreover, according to Zhou et al. [19], the essential oil obtained from *Eucalyptus grandis* of the Myrtaceae family has an excellent phytotoxic activity. Phytotoxicity is a biological phenomenon that affects the growth and development of plants, using secondary metabolites produced in nature. Among natural sources, EOs are strong candidates as they are sources of highly phytotoxic allelochemicals [20,21]. Allelochemicals are molecules that may have a natural origin and are considered important substances for the control of invasive plant species. In the Amazon, for example, two species of invasive plants can be found in management areas, *Mimosa pudica* and *Senna obtusifolia*, these species are described in the literature as species that can change the dynamics of the areas, as they exert interactions and competition with plants; in addition, they are plants that can cause damage to the oral mucosa of small and large ruminants and poisoning in these animals [22,23].

In addition, another property of pharmacological interest of the Myrtaceae family is its antioxidant capacity. The antioxidant properties of EOs from the Myrtaceae family are reported for some species [24]. Due to the high potential for scavenging free radicals, the search for new natural antioxidants has grown strongly, especially in view of the great pharmacological benefits arising from both the control of oxidative stress and its promising use in food preservation [25]. However, within the Myrtaceae family, there are species whose reports of their antioxidant and phytotoxic potential are unknown in the literature, such as *Calycolpus goetheanus* (Mart. ex DC.) O. Berg. The *Calycolpus* genus (Myrtaceae) has 15 species that are distributed in Central America until Minas Gerais (Brazil) and concentrated in northern South America [26], of those species, 10 occur in Brazil [27]. *Calycolpus goetheanus* (*ameixa-da-praia*, or “beach plum”), a shrub or tree species that bears edible fruits, native to Brazil, not endemic, occurring in the Amazon and the Brazilian Cerrado [28,29].

There are few reports on the chemical composition of *C. goetheanus* EOs. Studies carried out by [30] and [31], show the predominance of mono- and sesquiterpenes. Regarding the biological activities of *C. goetheanus*, there are no reports in the literature. In order to contribute to the scientific and economic knowledge of native plant species in the Amazon region, this study aimed to evaluate the chemical composition and phytotoxic and antioxidant potential of EOs from *C. goetheanus* specimens.

## 2. Results and Discussion

### 2.1. Yield

The highest yield of *C. goetheanus* essential oil obtained through hydrodistillation was from specimen B (1.10%), followed by specimen A (0.69%) and specimen C (0.20%). The yield obtained in specimen B’s essential oil was higher than that found in the essential oil of a sample collected in Maracana, State of Pará, Brazil, with a content equal to (1.0%) [30]. However, in the circadian study conducted by [31], with a specimen collected in Salvaterra on Marajó Island, State of Pará, Brazil, the contents were higher than those found in our study (1.2–2.3%).

### 2.2. Chemical Composition of the EOs

The chemical constituents identified in the EOs of the dried leaves of *C. goetheanus* specimens are listed in Table 1. In total, 103 compounds were identified and quantified through gas chromatography coupled to mass spectrometry GC-MS. 

The terpene class characterized the essential oil of *C. goetheanus* specimens. Specimen A was characterized by the presence of oxygenated sesquiterpenes (56.07%) and hydrocarbons (43.19%). Specimen B also showed high concentrations of oxygenated sesquiterpenes (43.83%) and hydrocarbons (43.38%), in addition to oxygenated monoterpenes (11.78%). The presence of oxygenated monoterpenes was also found in specimen A, but in low concentrations (0.77%). Specimen C was represented by hydrocarbon (60.17%) and oxygenated (39.83%) sesquiterpenes.

Specimen A had the oxygenated sesquiterpene β-eudesmol (22.83%) as its main compound, followed by (*E*)-caryophyllene (14.61), γ-eudesmol (13.87%), and bulnesol (8.09%). The oxygenated monoterpene 1,8-cineole (8.64%) and the hydrocarbon sesquiterpene (*E*)-Caryophyllene (5.86%) and δ-cadinene (5.78%) were the primary constituents of specimen B. Specimen C’s essential oil was characterized by the high concentration of the oxygenated sesquiterpene α-cadinol (9.03%) and the hydrocarbon sesquiterpenes δ-cadinene (8.01%), (*E*)-caryophyllene (6.74%), viridiflorene (6.7%), and germacrene D (6.34%).

Dos Santos et al. [31] evaluated the seasonal and circadian rhythms of the essential oil of a *C. goetheanus* specimen collected on Marajó Island (Pará) and obtained, as main constituents, 1,8-cineole (14.5–33.0%), followed by limonene (5.4–11.7%), δ-cadinene (0.0–9.9%), α-terpineol (3.5–7.9%), α-copaene (3.5–7.3%), and (*E*)-Caryophyllene (0.0–4.9%) in samples collected in the rainy (January) and dry (July) seasons, every 3 h (starting at 6 a.m. and ending at 9 p.m.). The limonene compound was not detected in any of the samples studied in this work, α-copaene was observed in the three studied specimens, but in low concentrations (0.97–2.92%). Furthermore, the α-terpineol compound was only identified in specimen B, with a low content (0.04%).

Pereira et al. [30] evaluated the chemical composition of a *C. goetheanus* sample collected in the municipality of Maracanã (Pará) and obtained, as main constituents, 1,8-cineole (44.75%), limonene (6.78%), α-terpineol (6.59%), and (*E*)-caryophyllene (6.26%). β-eudesmol and γ-eudesmol were the main constituents found in the essential oil of *C. goetheanus* specimen A, absent in the oil studied by [30]; however, they were observed, in low concentrations, in the samples studied by dos Santos, [31]. Germacrene D, one of the main constituents of the specimen C essential oil, and bulnesol from specimen A were not found in any of these works presented.

In addition, these identified compounds have potential for other applications, for example, 1,8-cineole is added to many cosmetic products due to its pleasant aroma and taste. This compound is reported in the literature as having several properties, such as: antioxidant, anti-inflammatory [34], insecticide [35], and antiproliferative [36]. (*E*)-Caryophyllene has a characteristic woody odor and is used in cosmetics and as food additives [37]. Its biological activities are widely reported in the literature and include antimicrobial [14], antiproliferative, and antiprotozoal [38]. There are also reports of its anticonvulsant [39], analgesic, and anti-inflammatory properties [40]. 

Germacrene D has antimicrobial [2,41] and cytotoxic [42] activities described in the literature. δ-Cadinene has acaricidal activity against *Psoroptes cuniculi* [43] and antimicrobial properties against the *Streptococcus pneumoniae* bacterium, the main etiological agent of respiratory infections [44]. Furthermore, this sesquiterpene has antiproliferative activity against human ovarian cancer cells (OVCAR-3) [45]. Oxygenated sesquiterpene β-eudesmol is reported to have cytotoxic activities against cells that cause cholangiocarcinoma or bile duct cancer [46,47]. 

### 2.3. Antioxidant Activity

The results of the ABTS^•+^ and DPPH^•^ radical scavenging assays were presented as Trolox Equivalent Antioxidant Capacity (TEAC) using Trolox as a reference standard. Additionally, to demonstrate the directly dependent values, the antioxidant activity was calculated from the equations of the straight line obtained from the standard (ABTS^•+^ y = 0.455x + 0.0002 R^2^ = 0.998; DPPH^•^ y = 0.2261x − 0.0094 R^2^ = 0.9831). Due to insufficient essential oil for specimen C, it was not possible to determine the antioxidant potential of this sample.

**Figure 1:**
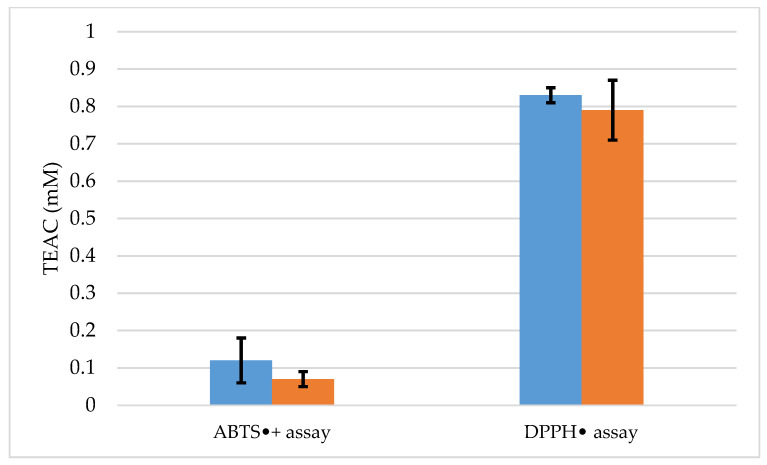
ABTS^•+^ and DPPH^•^ radical scavenging assay e Trolox equivalent antioxidant capacity de *Calycolpus goetheanus*. Values are expressed as mean and standard deviation (*n* = 3) of Trolox equivalent antioxidant capacity. The ABTS^•+^ and DPPH^•^ Radical Scavenging Assay (ABTS^•+^; DPPH^•^) 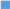 specimens B, and 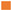 specimens A.

The DPPH^•^ assay values were 0.79 ± 0.08 and 0.83 ± 0.02 mM, respectively, for specimens A and B (Figure 1). The DPPH^•^ assay data confirm that both EOs from the specimens are active in the presence of the DPPH^•^ radical and have a good antioxidant capacity. On the other hand, ABTS^•+^ values were 0.07 ± 0.02 mM for specimen A and 0.12 ± 0.06 mM for specimen B. These results confirm that the antioxidant potentials of the samples were lower than the Trolox standard, and lower when compared to the DPPH assay. This difference is evident in both tests, as observed in other studies reported in the literature [48,49,50].

In the literature, there are no records of the antioxidant capacity of *C. goetheanus* EOs; however, the Myrtaceae family is described as having species with high antioxidant potential, as observed in studies [24,51]. In this sense, the antioxidant capacity shown by the *C. goetheanus* EOs may be associated with monoterpenes, and sesquiterpenic compounds 1,8-Cineole, (*E*)-caryophyllene, β-eudesmol, γ-eudesmol, δ-cadinene, and bulnesol, which are described in the literature for their antioxidant properties [52,53,54,55]. The high content of oxygenated sesquiterpenes shown for both EOs may have influenced the antioxidant potential of the samples, as these compounds can act individually or synergistically as antioxidants [56].

### 2.4. Phytotoxic Activity of the EOs

**Figure 2:**
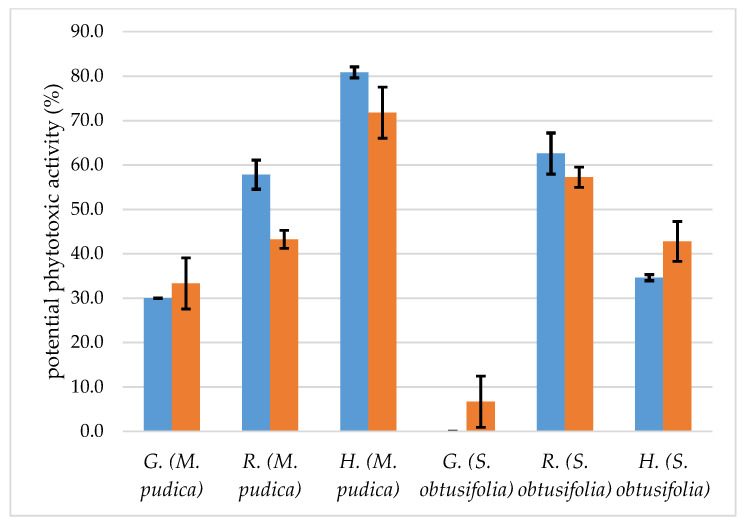
Potential phytotoxic activity of *C. goetheanus* EOs from *Calycolpus goetheanus*. 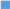 specimens B, and 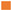 specimens C. G = germination, R = radicle, and H = hypocotyl.

The results of the potential phytotoxic effect of *C. goetheanus* specimens B, and C are shown in Figure 2. We found that the essential oil samples showed different levels of intensity of seed germination inhibition, both for *M. pudica* and *S. obtusifolia*; for example, the specimen C essential oil had inhibition values of 33.33 ± 5.77% and 6.67 ± 5.77%, for *M. pudica* and *S. obtusifolia*, respectively. With regard to the essential oil sample of specimen B, the percentage of inhibition was equal to 30.00 ± 0.00% for *M. pudica* and showed no phytotoxic effect on the germination of *S. obtusifolia*. Potential phytotoxic effects were more intense for receptor species *M. pudica*. In other works in the literature, it is demonstrated that this invasive plant species is more susceptible to damage caused by substances present in EOs [57,58]. 

Regarding radicle elongation, the intensity of inhibition varied according to the receptor species, the specimen C essential oil inhibited the radicle elongation of *M. pudica* by 43.24 ± 2.03% and *S. obtusifolia* by 57.24 ± 2.28%, showing a greater effect on *S. obtusifolia* (Figure 2). Specimen B’s essential oil had a greater inhibitory effect for the radicle elongation of *M. pudica* with an inhibition of 57.83 ± 3.28% and 62.57 ± 4.63 for *S. obtusifolia*. In both cases analyzed, we can see that, for this variable studied, receptor plant *S. obtusifolia* was the most affected by the EOs. The results of this study, when compared to the literature, do not follow the same pattern of response; for example, in previous studies, the most affected receptor species was *M. pudica* [59,60]; however, these response patterns depend on factors other than the receptor species, such as the chemical profile of the essential oil [60,61,62,63].

The effects of EOs from specimens B and C on hypocotyl elongation followed a different pattern from the effects on radicle elongation, and at a different intensity of inhibition, with receptor species *M. pudica* being the most affected by both essential oil samples, the inhibition values were 80.87 ± 1.25 and 71.78 ± 5.75%, respectively (Figure 2), while *S. obtusifolia* showed lower susceptibility to the effects of EOs, with intensity levels of 34.63 ± 0.69% and 42.79 ± 4.50%, respectively (Figure 2). In the literature, a minimum inhibition of 50% is considered a satisfactory standard to evaluate the potentials of an essential oil [64], which was partially observed in this work (Figure 2).

According to Shao et al., [65], the 1,8-cineole compound obtained lower results for the inhibition of the root growth of *Amaranthus retroflexus* and *Poa annua*, when compared to the other two major constituents of the *Seriphidium terrae-albae* essential oil (α-thujone and β-thujone). Other authors also demonstrate the phytotoxic potential of 1,8-cineole on different species of receptor plants [66,67,68]; in addition, compounds such as δ-cadinene and (*E*)-Caryophyllene have also shown phytotoxic potential on several plant species; in addition, other compounds such as δ-cadinene and (*E*)-Caryophyllene have also shown phytotoxic potential on several plant species such as *Mimosa pudica*, *Senna obtusifolia*, *Sinapis arvensis*, *Trifolium campestre*, and *Phalaris canariensis* weeds [58,69], results similar to those of other authors [63,70]. Jaradat [71] points out that the *Teucrium polium* L. essential oil has α-cadinol as the component with the highest content (46.80%). According to the author, the natural chemicals of this species are known for their phytotoxic effects against different types of invasive species. According to Elshamy et al. [72], the *Launaea spinosa* EOs, which have γ-eudesmol as the third highest component (6.31%), showed phytotoxic activity against *Portulaca oleracea*.

### 2.5. In Silico Study

**Figure 3:**
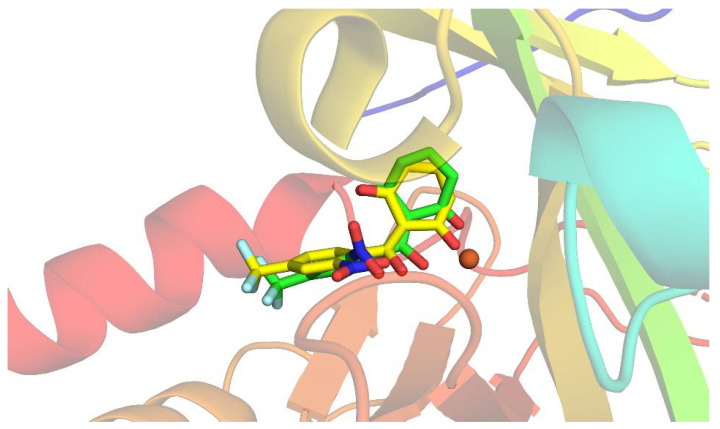
The structure obtained by redocking (yellow), overlapping the crystallographic structure (red) of HPPD complexed with NTBC.

In our results, specimens B and C showed good post-emergence herbicidal activity against the species *M. pudica* L. and *S. obtusifolia* (L.) Irwin and Barneby were used as weed models. The HPPD protein has been reported as the molecular target of substances that have post-emergence emergence herbicidal activity [73,74,75,76]. Therefore, we used this protein as a target in order to investigate the molecular interactions and the affinity energy formed in the HPPD-compounds complexes (Figure 3). According to the literature, the majority are those compounds that have a concentration above 5% of the substance in the essential oil [77,78,79,80,81].

To validate our docking protocol, we initially redocked the crystallographic ligand. For a docking protocol to be considered adequate, the RMSD value between the crystallographic ligand and the redocked ligand must be equal to or less than two angstroms [5,82,83,84].

**Figure 4:**
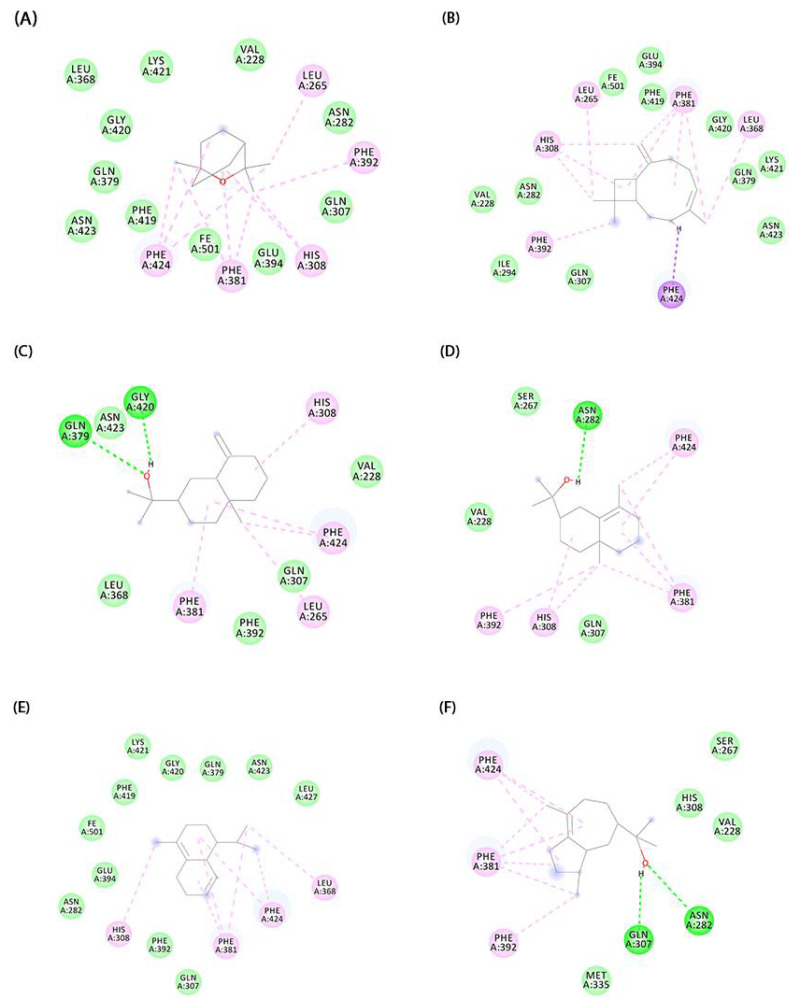
Docked conformation of molecules in the binding cavity of HPPD: In (**A**) we have the complex established with 1,8-Cineole, (**B**) (*E*)-Caryophyllene, (**C**) β-Eudesmol, (**D**) γ-Eudesmol, (**E**) δ-Cadinene, and (**F**) Bulnesol.

To perform the docking methodology, we first performed the crystallographic ligand redocking to assess whether the software is able to reproduce the mode of interaction observed in the crystallographic structure of the protein. For this, the NTBC present in the PDB 6J63 was redocked and the results of the fitting poses were evaluated considering the RMSD value and the fitting score. The RMSD value between the redocked ligand and the crystallographic one was 1.85 Å (Figure 4). This result proves that the docking protocol used is suitable for the investigation of the molecular binding of the investigated complex.

Then, molecular docking of the major compounds 2758 (1,8-cineole), 5281515 ((*E*)-Caryophyllene), 91457 (β-eudesmol), 6432005 (γ-eudesmol), 10657 (δ-cadinene), and 90785 (bulnesol) was performed. The affinity energy results are summarized in Table 2. In addition, the interactions established between the compounds and the HPPD active site are shown in Figure 4. From the simulated binding modes, it was possible to observe that the van der Waals and alkyl and pi-alkyl interactions were the main ones responsible for directing the receptor–ligand interaction. In some complexes, such as the one established between HPPD and α-cadinol, there was the formation of a hydrogen bond between Phe419 and the hydroxyl of the molecule. The difference in the mode of interaction and the distance between the ligands and Fe^2+^, present in the protein site, are capable of influencing the effectiveness of target inhibition [85,86]. Thus, the difference in EO inhibition capacity observed in phytotoxicity experiments may be related to the interaction of its major compounds with the protein site and its ability to chelate Fe^2+^.

## 3. Materials and Methods

### 3.1. Botanical Material

Aerial parts of three *C. goetheanus* specimens were collected in the coastal region of the State of Pará, in the city of Magalhães Barata, Brazil, the geographic coordinates of which are S 00°48′20.9′′ W 47°33′57.3′. *C. goetheanus* specimen (A) was collected on 4 October 2018 in a floodplain area on the left bank of the Curral River, and specimens (B) and (C) were collected on 20 September 2019, the first in a floodplain area on the right bank of the Curral River, while specimen (C) was collected in a secondary forest area (capoeira). The exsiccates were incorporated into the archive of Herbario Joao Murça Pires (MG) of Museu Paraense Emílio Goeldi, in the collection of Aromatic Plants of the Amazon, Belém, Pará and received records MG237476 (*C. goetheanus* A), MG237471 (*C. goetheanus* B), MG237475 (*C. goetheanus* C).

### 3.2. Preparation and Characterization of the Botanical Material

The samples of *C. goetheanus* leaves were dried in an oven with air circulation at 35 °C for 5 days, and then ground in a knife mill (Tecnal, model TE-631/3, Brazil). The moisture content was analyzed using an infrared moisture tester (ID50; GEHAKA, Duquesa de Goias, Real Parque, Sao Paulo, Brazil).

### 3.3. Extraction of EOs

The samples were subjected to hydrodistillation in modified Clevenger-type glass systems for 3 h, coupled to a refrigeration system to maintain the condensation water at around 12 °C. After the extraction, the oils were centrifuged for 5 min at 3000 rpm, de-hydrated with anhydrous sodium sulfate, and centrifuged again under the same conditions. Oil yield was calculated in mL/100 g. The oils were stored in amber glass ampoules, sealed with flame, and stored in a refrigerator at 5 °C [87].

### 3.4. Chemical Composition Analysis

The chemical compositions of the EOs of *C. goetheanus* (A, B, and C), were analyzed using a Shimadzu QP-2010 plus (Kyoto, Japan) a gas chromatography system equipped with an Rtx-5MS capillary column (30 m × 0.25 mm; 0.25 µm film thickness) (Restek Corporation, Bellefonte, PA, USA) coupled to a mass spectrometer (GC/MS) (Shimadzu, Kyoto, Japan). The program temperature was maintained at 60–240 °C at a rate of 3 °C/min, with an injector temperature of 250 °C, helium as the carrier gas (linear velocity of 32 cm/s, measured at 100 °C), and a splitless injection (1 μL of a 2:1000 hexane solution), using the same operating conditions as described in the literature [6,88,89,90]). The components were quantified using gas chromatography (GC) on a Shimadzu QP-2010 system (Kyoto, Japan), equipped with a flame ionization detector (FID) (Kyoto, Japan), under the same operating conditions as before, except for the carrier hydrogen gas. The retention index for all volatile constituents was calculated using a homologous series of *n*-alkanes (C_8_–C_40_) Sigma-Aldrich (St. Louis, MI, USA), according with Van den Dool and Kratz [91]. The components were identified by comparison (i) of the experimental mass spectra with those compiled in libraries (reference) and (ii) their retention indices to those found in the literature [32,33].

### 3.5. Trolox Equivalent Antioxidant Capacity (TEAC)

The ABTS^•+^ and DPPH^•^ assays were methods used for the assessment of the antioxidant capacities of EOs. The antioxidant potential of the studied substances was determined according to their equivalence to the potent antioxidant, Trolox (6-hydroxy-2,5,7,8-tetramethylchromono-2-carboxylic acid; Sigma-Aldrich; 23881-3; São Paulo, Brazil), and a water-soluble synthetic vitamin E analogue.

#### 3.5.1. The ABTS^•+^ Radical Scavenging Assay 

The ABTS^•+^ Assay was determined according to the methodology adapted from Miller et al. [92], and modified by Re et al. [93]. ABTS^•+^ (2,2′-Azino-bis (3-ethylbenzothiazoline-6-sulfonic acid); Sigma-Aldrich; A1888; São Paulo, Brazil) was prepared using 7 mM ABTS^•+^ and 140 mM of potassium persulfate (K_2_O_8_S_2_; Sigma Aldrich; 216224; São Paulo, Brazil) incubated at room temperature without light for 16 h. Then, the solution was diluted with phosphate-buffered saline until it reached an absorbance of 0.700 (± 0.02) at 734 nm.

To measure the antioxidant capacity, 2.97 mL of the ABTS^•+^ solution was transferred to the cuvette, and the absorbance at 734 nm was determined using a Biospectro SP 22 spectrophotometer (São Paulo, Brazil). Then, 0.03 mL of the sample was added to the cuvette containing the ABTS^•+^ radical and, after 5 min, the second reading was performed. The synthetic antioxidant Trolox (6-hydroxy-2,5,7,8-tetramethylchromono-2-carboxylic acid; Sigma Aldrich; 23881-3; São Paulo, Brazil) was used as a standard solution for the calibration curve (y = 0.455x + 0.0002, where y represents the value of absorbance and x, the value of concentration, expressed as mM; R^2^ = 0.998). The results were expressed as mM. The values found for the samples were compared to the Trolox standard (1 mM).

#### 3.5.2. DPPH^•^ Radical Scavenging Assay

The test was carried out according to the method proposed by [94] To measure the antioxidant capacity, initially, the absorbance of DPPH^•^ solution (2,2-diphenyl-1-picrylhydrazyl; Sigma-Aldrich; D9132; São Paulo, Brazil) 0.1 mM diluted in ethanol was determined. Subsequently, 0.6 mL of DPPH^•^ solution, 0.35 mL of distilled water, and 0.05 mL of the sample were mixed and placed in a water bath at 37 °C for 30 min. Thereafter, the absorbances were determined in a spectrophotometer Bioespectro SP 22 (São Paulo, Brazil) at 517 nm. The synthetic antioxidant Trolox (6-hydroxy-2,5,7,8-tetramethylchromono-2-carboxylic acid; Sigma-Aldrich; 23881-3; São Paulo, Brazil) was used as a standard solution for the calibration curve (y = 0.2261x − 0.0094, where y represents the value of absorbance and x, the value of concentration, ex-pressed as mM; R^2^ = 0.9831). The results were expressed as mM. The values found for the samples were compared to the Trolox standard (1 mM).

### 3.6. Phytotoxic Potential Activity of the EOs

The phytotoxic potential bioassays, on the two species of receptor plants M. *pudica* and *S. obtusifolia*, were carried out with EOs of *C. goetheanus*, with only the EOs whose yield was ≥0.5 mL, that is, the samples A, and B, please, see Supplemental Material.

#### 3.6.1. Seed Treatment

The phytotoxic activities were developed at the Agroindustry Laboratory of EM-BRAPA Amazonia Oriental, Belém, Pará, Brazil. The phytotoxic activity was evaluated in two bioindicator species that are weeds of common pasture areas in the Amazon region: *M. pudica* L. and *S. obtusifolia* (L.) Irwin and Barneby. Phytotoxic effects were analyzed on different parameters: percentage of seed germination and radicle and hypocotyl elongation. The seeds were collected in areas of cultivated pastures, in the degradation phase, in the municipality of Terra Alta-PA, underwent a cleaning process, and were treated in order to break dormancy, via immersion in concentrated sulfuric acid for 15 min [57,58].

#### 3.6.2. Germination

The bioassays were performed as proposed by [57,58] with adaptations, in a BOD-type chamber, with controlled conditions of 25 °C and a photoperiod of 12 h, with monitoring for three days, daily counts, and elimination of germinated seeds. Seeds with a root length of 2 mm were considered germinated. 

Each 9.00 cm diameter Petri dish was lined with a sheet of qualitative filter paper, where the test solutions were added only once, at the beginning of the bioassays, using 3 mL of the test solutions diluted in n-hexane. After the total evaporation of the solvent, 2.5 mL of distilled water was added; later, 10 seeds of the two receptor species (*M. pudica*, and *S. obtusifolia*) were added to each dish; the procedure was performed in triplicate. 

#### 3.6.3. Radicle and Hypocotyl Elongation

The radicle and hypocotyl elongation were performed in BOD-type chambers with a constant temperature of 25 °C and a photoperiod of 24 h. Each 9.0 cm diameter Petri dish received 3.0 mL of the test solution, lined with filter paper. The EOs were tested at the same concentrations as the germination bioassays. After evaporation of the solvent, a volume of 3 mL of distilled water was added, thus maintaining the original concentration. 

The test solutions were added only once, at the beginning of the bioassays, and from then on only distilled water was added, whenever it was required to maintain the seedlings. In each of the plots, three pre-germinated seeds were placed for three days. At the end of the 7-day growth period, the length of the radicle and hypocotyl was measured. The control treatment consisted only of using distilled water. For all bioassays, the EOs were tested at concentrations of 1.0% (*v*/*v*), and the bioassays were performed in triplicate.

### 3.7. Prediction of Molecular Interactions 

#### Molecular Docking

The protein 4-hydroxyphenylpyruvate dioxygenase (HPPD) has been reported as a molecular target for compounds with post-emergence herbicidal activity [73,74,75,76]. Because of this, we used this protein as a target for the major compounds in *C. goetheanus* essential oil. The three-dimensional structure of the HPPD protein was collected from the Protein Data Bank from PDB ID 6J63 [73]. The substances used in our studies were collected in PubChem from the CID’s compounds: 2758 (1,8-Cineole), 5281515 ((*E*)-Caryophyllene), 91457 (β-Eudesmol), 6432005 (γ-Eudesmol), 10657 (δ-Cadinene), and 90785 (Bulnesol). The molecular structure of these compounds was optimized with B3LYP/6-31G [95,96] using the Gaussian 09 [97]. To evaluate the molecular binding mode, the Molegro Virtual Docker 5.5 software [98,99,100] was used. The MolDock Score (GRID) scoring function was used with a Grid resolution of 0.30 Å. The protein binding site has a cavity with a volume of 388,096 and a surface of 1,076,482. The center is at X: 26.16, Y: −22.87, and Z: 4.28. The radius used to encompass the pocket binding was 12 Å. The MolDock SE algorithm was used for docking with the number of runs equal to 10, 1500 max interactions, and a max population size equal to 50. The maximum evaluation of 300 steps with a neighbor distance factor equal to 1 and energy threshold equal to 100 was used during the molecular docking simulation.

## 4. Conclusions

The chemical profile of EOs was characterized by the high content of hydrocarbon sesquiterpenes, especially (*E*)-caryophyllene (4.86 ± 13.61%), germacrene D (0.64 ± 6.34%), δ-cadinene (4.69 ± 8.01%), and oxygenated sesquiterpenes, mainly γ-eudesmol (1.56 ± 12.87%), α-cadinol (9.03%), and epi-α-muurolol, (5.69%). This significant sesquiterpene content may have influenced the strong elimination capacity of DPPH^•^ free radicals observed in the EO of specimens A (0.79 ± 0.08 mM) and B (0.83 ± 0.02 mM) of *C. goetheanus*.

The recipient species *Mimosa pudica* presented greater sensitivity to the EOs of specimens B and C, with higher phytotoxic potential in hypocholic elongation with 80.87 ± 1.25% (specimen B) and 71.78 ± 5.75% (specimen C). This high inhibition potential may be peated to the presence of some terpenic compounds, such as δ-cadinene, 1,8-cineole, and (*E*)-Caryophyllene. 

In the in silico study, specimens B and C showed good herbicide activity against the species *M. pudica* L. and *S. obtusifolia*, which may be associated with the difference in the inhibition capacity of the OE observed in the phytotoxicity experiments through the interaction of their major compounds with the protein site.

## Figures and Tables

**Table 1:** Chemical composition of EOs isolated from *Calycolpus goetheanus* leaves through hydrodistillation. The concentration values of the compounds are relative to the percentage (%).

RI_L_	RI_C_	Constituents	Specimen		
			A (%)	B (%)	C (%)
932	933	α-Pinene		0.33	
988	990	Myrcene		0.17	
1014	1016	α-Terpinene		0.08	
1020	1024	ρ-Cymene		0.03	
1026	1033	1,8-Cineole		8.64	
1044	1046	(*E*)-β-Ocimene		0.03	
1054	1057	γ-Terpinene		0.28	
1086	1088	Terpinolene		0.09	
1095	1099	Linalool	0.77	0.36	
1162	1166	δ-Terpineol		0.04	
1174	1177	Terpinen-4-ol		0.24	
1186	1192	α-Terpineol		2.5	
1335	1340	δ-Elemene			2.91
1345	1352	α-Cubebene	0.16	0.91	
1369	1369	Cyclosativene	0.16	0.07	
1373	1374	α-Ylangene	0.31	0.06	0.04
1374	1379	α-Copaene	0.97	2.53	2.92
1390	1393	Sativene		0.11	
1389	1394	β-Elemene			2.71
1400	1398	β-Longipinene		0.04	
1409	1414	α-Gurjunene	0.17	2.24	0.24
1417	1426	(*E*)-Caryophyllene	14.61	5.86	6.74
1421	1428	β-Duprezianene			0.01
1430	1432	β-Copaene	0.19	0.27	0.74
1434	1442	γ-Elemene	0.14		2.91
1439	1442	Aromadendrene		0.52	0.24
1442	1446	6,9-Guaiadiene			0.47
1448	1449	*cis*-Muurola-3,5-diene	0.77	0.02	
1451	1454	*trans*-Muurola-3,5-diene	0.61	0.87	0.53
1452	1458	α-Humulene	1.85	2.35	4.73
1458	1459	*allo*-Aromadendrene	0.66		0.24
1464	1465	9-*epi*-(*E*)-Caryophyllene		1.17	0.12
1471	1473	4,5-*di*-*epi*-aristolochene		0.11	
1475	1478	*trans*-Cadina-1(6),4-diene		3.83	
1478	1480	γ-Muurolene			1.33
1471	1483	Dauca-5,8-diene	1.82		0.58
1483	1484	α-Amorphene	0.4	0.22	
1484	1486	Germacrene D	0.64		6.34
1492	1488	*cis*-β-Guaiene	0.6	0.23	
1489	1491	β-Selinene		3.21	2.48
1492	1494	δ-Selinene			1.99
1493	1496	*trans*-Muurola-4(14),5-diene		0.62	
1498	1500	α-Selinene		2.89	
1500	1504	α-Muurolene	0.89	2.91	2.11
1503	1505	β-Dihydro agarofuran		0.93	
1496	1506	Viridiflorene	1.7		6.7
1501	1509	Epizonarene	0.75		
1513	1518	γ-Cadinene	1.07	1.01	0.65
1511	1518	δ- Amorphene	2.31		1.13
1514	1521	β-Curcumene	0.12		
1520	1522	7-*epi*-α-Selinene		0.15	0.49
1521	1528	*trans*-Calamenene		0.63	
1522	1531	δ-Cadinene	5.69	5.78	8.01
1528	1533	Zonarene	2.73	1.35	
1533	1538	*trans*-Cadina-1,4-diene	0.81	1.77	0.51
1532	1539	γ-Cuprene			0.17
1537	1542	α-Cadinene		0.47	0.53
1540	1546	Selina-4(15),7(11)-diene	2.01		
1544	1547	α-Calacorene	0.49	1.05	0.1
1548	1551	α-Agarofuran		0.04	
1448	1552	Elemol			0.38
1545	1552	Selina-3,7(11)-diene	1		0.24
1447	1556	Italicene epoxide		0.02	
1562	1559	*epi*-Longipinanol			0.07
1559	1563	Germacrene B		0.11	1.26
1561	1568	(*E*)-Nerolidol	1.93	1.23	
1567	1575	Palustrol		4.97	1.09
1577	1581	Spathulenol			1.34
1570	1581	Dendrolasin		0.13	
1582	1585	Caryophyllene oxide		0.08	
1586	1589	Gleenol		2.46	
1586	1595	Thujopsan-2-α–ol	0.52		
1590	1598	Globulol	0.43		4.02
1592	1598	Viridiflorol	0.36	2.58	3.68
1600	1606	Rosifoliol			0.91
1602	1611	Ledol	0.58	3.6	
1608	1613	Humulene epoxide II			0.2
1607	1620	5-*epi*-7-*epi*-α-Eudesmol	0.16		
1618	1623	Junenol			0.94
1618	1626	1,10-*di-epi*-Cubenol	0.41		
1622	1628	10-*epi*-γ-Eudesmol		4.81	
1629	1630	Eremoligenol	0.41		2.57
1630	1633	γ-Eudesmol	13.87	3.33	1.56
1630	1633	Muurola-4,10(14)-dien-1-β-ol			5.31
1627	1637	1-*epi*-Cubenol		3.3	
1640	1639	Hinesol	0.94	2.16	
1640	1647	*epi*-α-Muurolol			5.69
1645	1650	Cubenol	1.81	4.03	
1644	1655	α-Muurolol		1.63	1.62
1652	1659	α-Eudesmol		2.79	
1652	1660	α-Cadinol			9.03
1656	1663	Valerianol	3	3.98	
1658	1664	Selin-11-en-4-α-ol		0.19	0.54
1662	1667	7-*epi*-α-Eudesmol		0.64	
1658	1667	*neo*-Intermedeol			0.12
1649	1667	β-Eudesmol	22.83		
1670	1669	Bulnesol	8.09		
1665	1670	Intermedeol			0.16
1679	1673	Khusinol		0.28	
1675	1679	Cadalene		0.12	
1685	1687	α-Bisabolol		0.52	
1700	1708	Eudesm-7(11)-en-4-ol	0.73		0.6
1702	1715	10-*nor*-Calamenen-10-one		0.03	
		Hydrocarbon monoterpenes		1.01	
		Oxygenated Monoterpenes	0.77	11.78	
		Hydrocarbons sesquiterpenes	43.16	43.38	60.17
		Oxygenated Sesquiterpenes	56.07	43.83	39.83
		Total	100	100	100

IR_C_: calculated from a series of n-alkanes (C_8_–C_40_) in a DB-5MS column capillary column, IR_L_: [32,33]; IR_C_: calculated retention index; IR_L_: literature retention index.

**Table 2:** Moldock scores obtained from the docking protocol using Molegro Virtual Docker 5.5.

Molecule	MolDock Score	Rerank Score
1,8-Cineole	−37.63	−33.03
(*E*)-Caryophyllene	−81.15	−63.10
β-Eudesmol	−73.23	−55.07
γ-Eudesmol	−72.77	−63.24
δ-Cadinene	−63.73	−53.31
Bulnesol	−85.15	−68.20

## Data Availability

Not applicable.

## Supplementary Materials

The following supporting information can be downloaded at: https://www.mdpi.com/article/10.3390/molecules27154678/s1, Experimental phytotoxicity tests of EOs, Figure S1.

## Sample Availability

Samples of the compounds *Calycolpus gotheanus* are available from the authors.
