# Supplementary material for: Phytochemical Profile and Herbicidal (Phytotoxic), Antioxidants Potential of Essential Oils from Calycolpus goetheanus (Myrtaceae) Specimens, and in Silico Study
Source: Molecules. 2022 Jul 22;27(15):4678. doi: 10.3390/molecules27154678 (PMC9331371; doi:10.3390/molecules27154678)
Supplement: Supplementary file 1 [file molecules-27-04678-s001.zip › molecules-1799801-supplementary.pdf]

molecules

MDPI

Article

Phytochemical Profile and Herbicidal (Phytotoxic), Antioxidants Potential of Essential Oils from *Calycolpus goteanus* (Myrtaceae) Specimens, and in Silico Study

Celeste de Jesus Pereira Franco [1], Oberdan Oliveira Ferreira [2], Jorddy Neves Cruz [3], Everton Luiz Pompeu Varela [2,4], Ângelo Antônio Barbosa de Moraes [1], Lidiane Diniz do Nascimento [5], Márcia Moraes Cascaes [6], Antônio Pedro da Silva Souza Filho [7], Rafael Rodrigues Lima [3], Sandro Percário [4], Mozaniel Santana de Oliveira [5,*] and Eloisa Helena de Aguiar Andrade [2,5,6]

[1] Faculdade de Química, Universidade Federal do Pará, Rua Augusto Corrêa S/N, Guamá, 66075-900 Belém, Pará, Brazil; celeste.frango12@gmail.com (C.d.J.P.F.); angeloquimica17@gmail.com (Â.A.B.d.M.)
[2] Programa de Pós-Graduação em Biodiversidade e Biotecnologia-Rede Bionorte, Instituto de Ciências Biológicas, Universidade Federal do Pará, Rua Augusto Corrêa S/N, Guamá, 66075-900 Belém, Pará, Brazil; oberdan@museu-goeldi.br (O.O.F.); evertonlpvalerla@gmail.com (E.L.P.V.); eloisa@museu-goeldi.br (E.H.d.A.A.)
[3] Laboratory of Functional and Structural Biology, Institute of Biological Sciences, Universidade Federal do Pará, Rua Augusto Corrêa S/N, Guamá, 66075-900, Belém, Pará Brazil; jorddy.cruz@icb.ufpa.br (J.N.C.); rafalima@ufpa.br (R.R.L.)
[4] Laboratório de Pesquisas em Estresse Oxidativo, Instituto de Ciências Biológicas, Universidade Federal do Pará, Rua Augusto Corrêa S/N, Guamá, 66075-900 Belém, Pará, Brazil; percario@ufpa.br (S.P.)
[5] Laboratório Adolpho Ducke—Coordenação de Botânica, Museu Paraense Emílio Goeldi, Av. Perimetral, 1901, Terra Firme, 66077-830, Belém, PA, Brasil, Brazil; lidianenascimento@museu-goeldi.br (L.D.d.N.)
[6] Programa de Pós-Graduação em Química, Universidade Federal do Pará, Rua Augusto Corrêa S/N, Guamá, 66075-900 Belém, Pará, Brazil; cascaesmm@gmail.com (M.M.C.)
[7] Embrapa Amazônia Oriental, Tv. Dr. Enéas Pinheiro, s/n-Marco, 66095-903 Belém, Pará, Brazil; antoniopedro.filho@embrapa.br
* Correspondence: mozaniel.oliveira@yahoo.com.br or mozaniel.oliveira@museu.goeldi.br

Citation: Franco, C.d.J.P.; Ferreira, O.O.; Cruz, J.N.; Varela, E.L.P.; de Moraes, Â.A.B.; Nascimento, L.D.d.; Cascaes, M.M.; Souza Filho, A.P.d.S.; Lima, R.R.; Percário, S.; et al. Phytochemical Profile and Herbicidal (Phytotoxic), Antioxidants Potential of Essential Oils from *Calycolpus goteanus* (Myrtaceae) Specimens, and in Silico Study. *Molecules* **2022**, *27*, 4678. https://doi.org/10.3390/molecules27154678

Academic Editors: Henryk H. Jeleń and Antoni Szumny

Received: 17 June 2022
Accepted: 20 July 2022
Published: 22 July 2022

Publisher's Note: MDPI stays neutral with regard to jurisdictional claims in published maps and institutional affiliations.

Copyright: © 2022 by the authors. Licensee MDPI, Basel, Switzerland. This article is an open access article distributed under the terms and conditions of the Creative Commons Attribution (CC BY) license (https://creativecommons.org/licenses/by/4.0/).

Abstract: The essential oil (EO) of Calycolpus goetheanus (Myrtaceae) specimens (A, B, and C) were obtained through hydrodistillation. The analysis of the chemical composition of the EOs was by gas chromatography coupled with mass spectrometry CG-MS, and gas chromatography coupled with a flame ionization detector CG-FID. The phytotoxic activity of those EOs was evaluated against two weed species from common pasture areas in the Amazon region: *Mimosa pudica* L. and *Senna obtusifolia* (L.) The antioxidant capacity of the EOs was determined by (DPPH•) and (ABTS•+). Using molecular docking, we evaluated the interaction mode of the major EO compounds with the molecular binding protein 4-hydroxyphenylpyruvate dioxygenase (HPPD). The EO of specimen A was characterized by β-eudesmol (22.83%), (*E*)-caryophyllene (14.61%), and γ-eudesmol (13.87%), while compounds 1,8-cineole (8.64%), (*E*)-caryophyllene (5.86%), δ-cadinene (5.78%), and palustrol (4.97%) characterize the chemical profile of specimen B's EOs, and specimen C had α-cadinol (9.03%), δ-cadinene (8.01%), and (*E*)-caryophyllene (6.74%) as the majority. The phytotoxic potential of the EOs was observed in the receptor species *M. pudica* with percentages of inhibition of 30%, and 33.33% for specimens B and C, respectively. The EOs' antioxidant in DPPH• was 0.79 ± 0.08 and 0.83 ± 0.02 mM for specimens A and B, respectively. In the TEAC, was 0.07 ± 0.02 mM for specimen A and 0.12 ± 0.06 mM for specimen B. In the results of the in silico study, we observed that the van der Waals and hydrophobic interactions of the alkyl and pi-alkyl types were the main interactions responsible for the formation of the receptor–ligand complex.

Keywords: natural products; volatile compounds; terpenes; allelopathy; antioxidant capacity

Supplementary material S1

Experiments on the phytotoxic potential of essential oils from *Calycolpus goetheanus*.

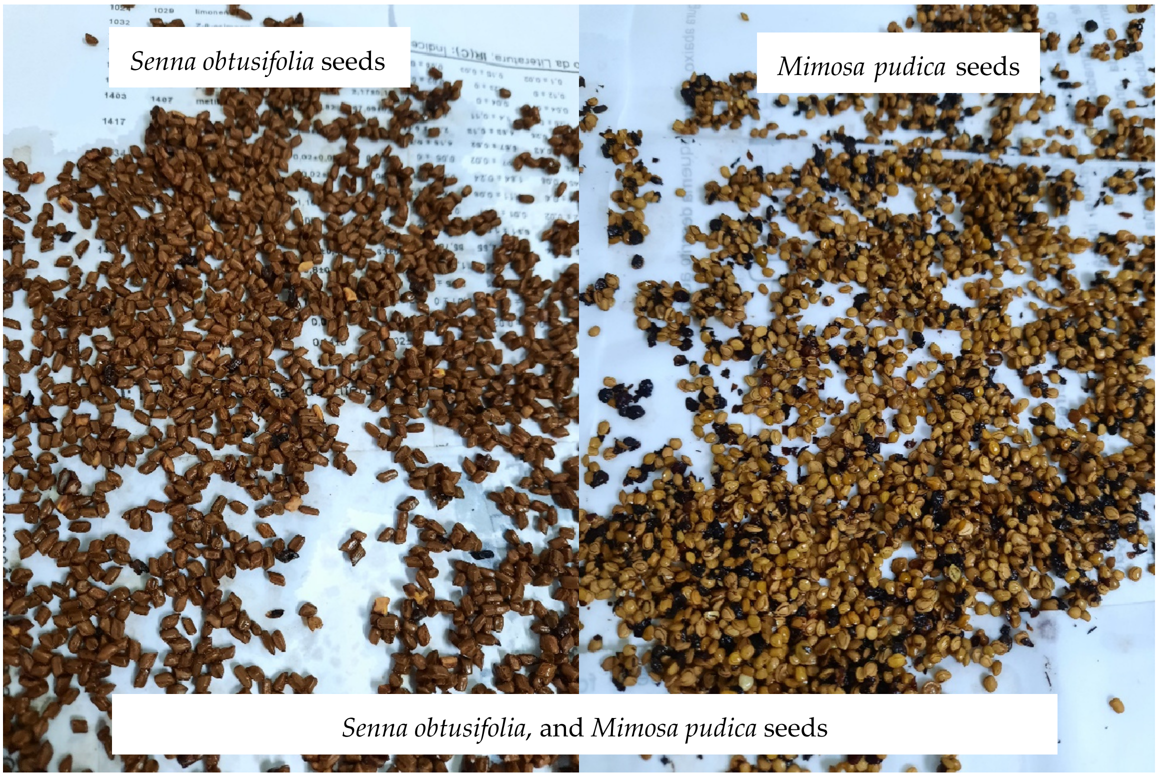

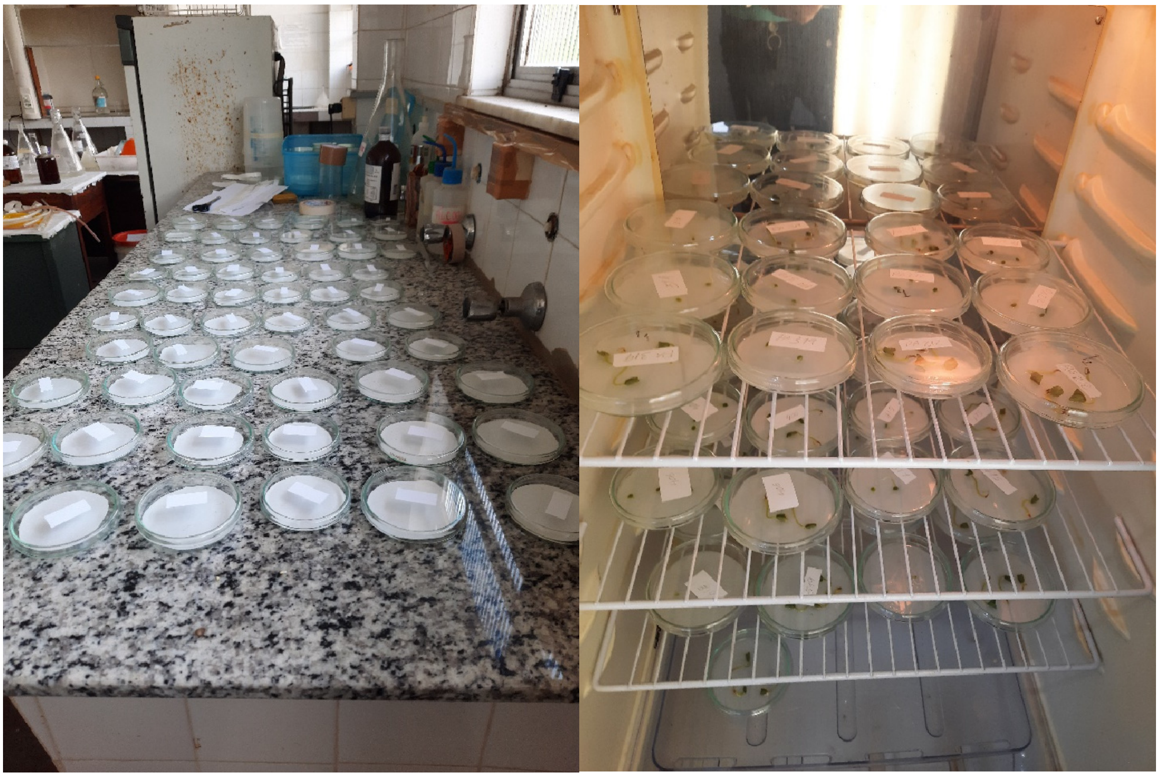

Senna obtusifolia seed germination

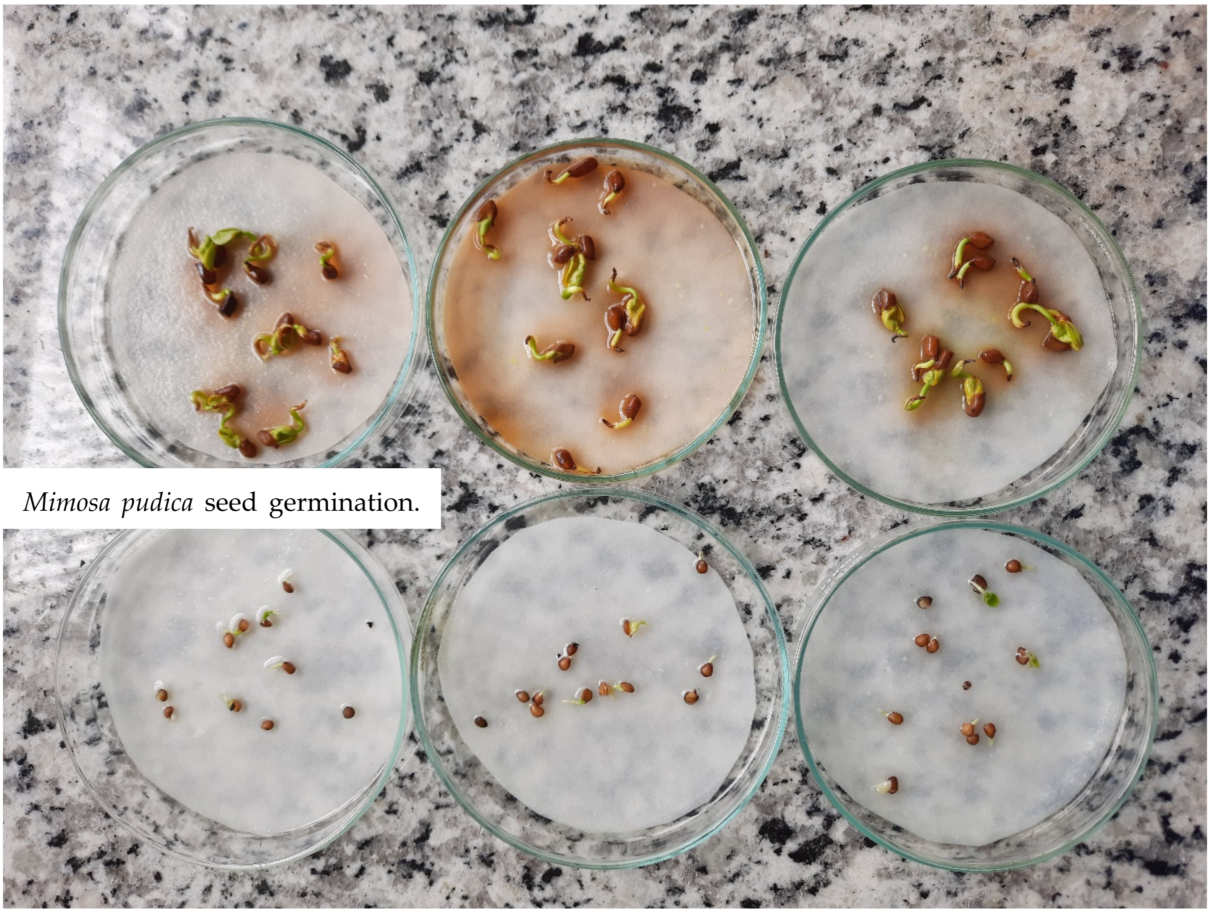

Mimosa pudica seed germination.

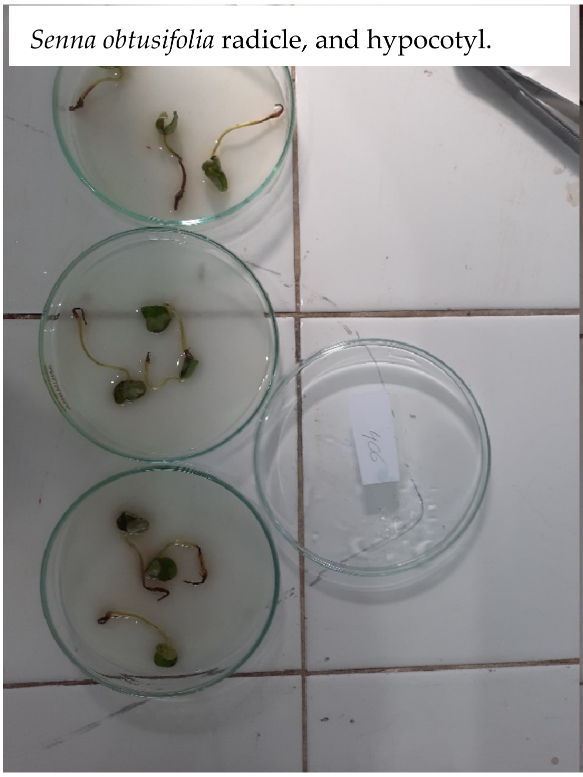

Senna obtusifolia radicle, and hypocotyl.

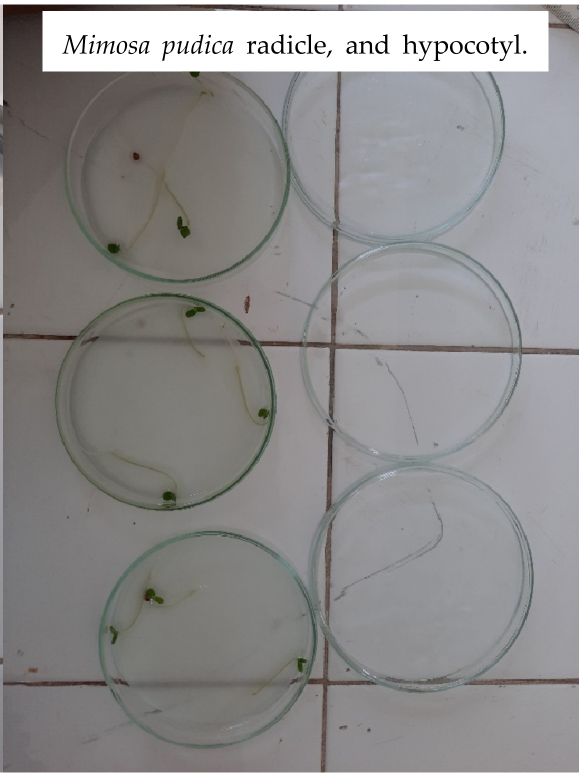

Mimosa pudica radicle, and hypocotyl.

Seed germination

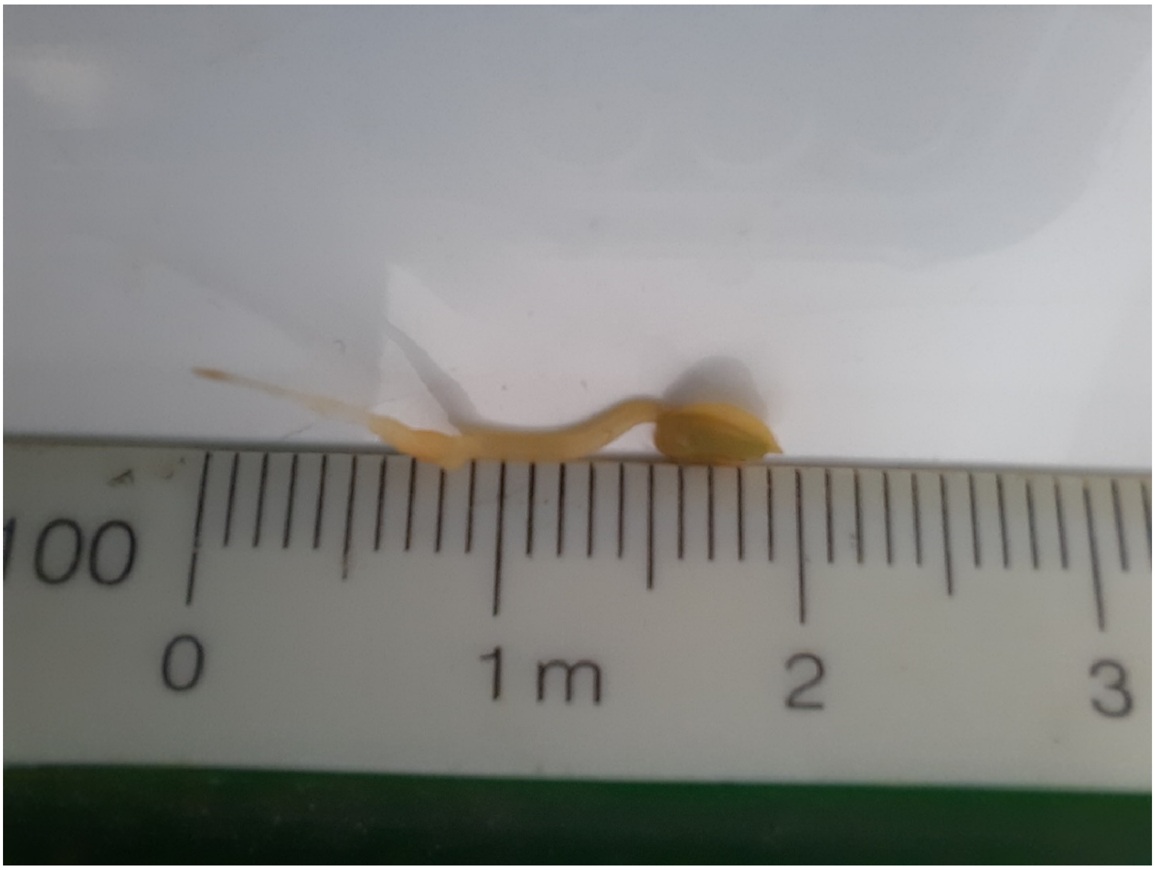
100
0
1m
2
3
